# “You’re Not Born with Talent” Talented Girls’ and Boys’ Perceptions of Their Talents as Football Players

**DOI:** 10.3390/sports4010006

**Published:** 2016-01-27

**Authors:** Stig Arve Sæther, Ingar Mehus

**Affiliations:** Department of Sociology and Political Science, Norwegian University of Science and Technology, Dragvoll, Trondheim 7491, Norway; ingar.mehus@svt.ntnu.no

**Keywords:** football, talent development, youth sports, skills, perception of talent

## Abstract

Generally in sports, there is a strong assumption of a connection between skill level in young age and adulthood. Studies have mainly focused on the coaches’ understanding and role in identifying and developing talent. In this article we turn our attention towards the athletes’ perspectives, interviewing talented young football players (five boys and five girls) about their perceptions of their own talent and development. The objective of the article is to investigate how boys and girls perceive their talent and to discuss how various perceptions influence coaching practice in talent development. We introduce the following questions: (a) do the players use a static or dynamic perception of their own talent and (b) do the players consider specific or general skills to be most important in their skill development? Results show that the boys have a more static perception of talent compared to the girls. Furthermore, the boys in this study stress the importance of highly specified skills. The girls have a more balanced view on what is important, but tend to stress the importance of basic skills. The study suggests two potential implications. First, the coaches should be aware of the possible vulnerability following players’ static perception of talent. Second, an exclusive focus on specified skills might make for less optimal preparation for the changing demands young players meet when moving through the different levels of play on their way to high level football. In future research it would be interesting to investigate how players with a lower skill level, not yet regarded as talent, perceive their talent and skill development.

## 1. Introduction

Most top-level clubs in European football are looking for the most talented boys and girls. The players who, compared with their peers, have skills that are recognized and highlighted as important and are described as potential professional football players [1]. The value and reasoning for the clubs’ and coaches’ focus on identifying the most talented players rest on several basic assumptions regarding talent development. Some well-established assumptions are that talent is hereditary, that talent is domain-specific, that a trained eye can identify talent at an early age, and that such early indicators predict future success [2].

Talented football players are obviously part of selection systems where the benefits of being among the selected are clear [3], even though there are many obstacles in this process [4]. However, focusing on identifying and selecting the most talented players could impact both the players’ assessments of their own skills, and which skills they think are preferable for being defined as talented players. From a player perspective, they are dependent on understanding which skills to develop to increase the likelihood of becoming a professional football player. They are confronted with many of the basic assumptions that underlie the selection process, coloring their understanding of the value of their own talent. Abbott and Collins [5] distinguish between static and dynamic perception of skill development. The static perception focuses on skills as congenital and less trainable. This perspective basically focuses on identifying the most talented players, where the players must prove their skills to the coaches and clubs. The dynamic perception highlights skills to be largely dependent on efforts to improve, and sees talent as something players achieve through training. It should be noted that talent identification and talent development are not necessarily experienced as separate processes for all football coaches, but rather are reflexively aligned [6]. Interestingly though, research has shown that top-level coaches have problems defining the criteria used when identifying talented players [7].

## 2. Context

Even if previous research on factors affecting talent development has been extensive among male players [8,9,10], research has not been able to confirm most of the basic assumptions [8,11]. Research investigating female players’ talent development has been less extensive, with a lack of research targeting the development process from a longitudinal perspective [12,13]. A factor indicating that the selection process among female players is less essential compared to male players is the less pronounced relative age effect [14]. This effect is described as the tendency of selecting talented players by an early birth month, especially the first three months of the year [15,16,17]. These results could indicate that female players could regard their skills as less dependent on talent, which could suggest that they will lean on a more dynamic perception of talent.

Partly based on the basic assumptions of talent development, there are different models and theoretical frameworks aiming to understand talent development. The focus on training volume has been highlighted [18], introducing the term deliberate practice, indicating a linear relationship between training volume and skill level. Deliberate practice is characterized by being controlled by a coach, giving instructions and guidelines, as well as the training session having clear goals. This perspective has, however, been challenged by Côté and Fraser-Thomas [19], introducing deliberate play as an alternative to deliberate practice, where deliberate play is characterized by being self-organized and does not necessarily include a clear goal for every training session. These two perspectives on talent development also indicate the difference between focusing on highly specific skills compared to more general football skills. While some players tend to focus on developing highly specific skills, others focus on more general skills, compensating for deficiencies in one area by strengthening others [20]. The complexity of developing expertise in football is an argument for not needing extraordinary capacities within all of the various technical, psychological and physical demands [21]. Even so, Haugaasen and Jordet [22] highlighted that football-specific practice is essential in talent development.

The coach is of great importance for the development of young players [23], and coaches’ understanding of player development impacts the players’ development [24,25]. If one accepts the strong focus on the identification and selective targeting of the few “chosen” talented players as a strong paradigm in football, one can also imagine that the players’ perceptions of their own talent is essential for their own development. In spite of this paradigm, previous research has, to a small extent, studied how talented footballers perceive their own talent and what factors have made an impact on their development.

### The Current Study

This study is designed as a focus group interview with talented football players in order to investigate their perceptions of talent and how their development has been dependent on their own talent. The objective of the article is to investigate how boys and girls perceive their talent and to discuss how various perceptions influence coaching practice in talent development. We introduce the following questions: (a) do the players use a static or dynamic perception of their own talent and (b) do the players consider specific or general skills most important in their skill development?

## 3. Method

### 3.1. Participants

The respondents are a selection of 10 Norwegian players (five boys and five girls) aged 14–16 years. Participants were recruited to participate in this study if they met the following inclusion criteria: Participants must have been selected to a district, region or age-specific national team, within the last 12 months. Representatives from the regional football association selected the players.

### 3.2. Data Collection Procedure

The first author was the moderator of two focus group interviews in September (three girls and one boy) and November 2013 (four boys and two girls). Focus group interviews were conducted in the office of the local football association, with chairs arranged in a circular manner around a boardroom table. A tape recorder was set up in the center of the table. The two sessions lasted 37 and 45 min.

Focus groups have proven valuable in similar settings, involving Danish talented young football players [26]. Focus groups as a method for data collection emphasize the relatively “non-hierarchical” relationship between moderator and participants [27], encouraging an informal atmosphere in which participants speak freely about all included topics without straying from the subject [28]. Another major advantage is that focus groups allow researchers to observe interactions and discussions. Statements and arguments from participants in the group could be analyzed through consensus and dissensus [29]. According to Berg and Lune [28], some important disadvantages of focus groups are: (a) only group, not individual, responses are obtained in the results; (b) dominant personalities may overpower and steer the group’s responses unless the moderator is sufficiently active; and (c) focus group data does not offer the same depth of information as a long semi-structured interview. Hence, the moderator’s guide was made with the purpose of providing players with the opportunity to elaborate on the various topics that were raised, while the moderator had the opportunity to follow up “tracks” in the conversation. All players were systematically asked to answer all questions, which they did, ensuring that all players got to speak their opinions on the matter. Examples of questions from the moderators guide include: “is talent trainable?”, “do you consider specific skills more important than general skills?” and “what are your thoughts on explaining how you have reach this skill level?”.

### 3.3. Analytical Process

The interviews were transcribed verbatim, analyzed through content analysis and coded in two categories: perceived talent and general *vs.* specific skills. The data material was sorted by these two categories, searching for patterns of consensus and dissensus. During the focus group interviews the moderator took notes of interactionary cues including headshakes, interrupting each other’s sentences and side comments. These observations were also coded and used as a supplement in our analysis.

## 4. Results and Discussion

Results show that the boys have a more static perception of talent compared to the girls. Furthermore, the boys in this study stress the importance of highly specified skills. The girls have a more balanced view on what is important, but tend to stress the importance of basic skills.

### 4.1. The Girls’ and Boys’ Understandings of Talent

The girls and boys in this study have already been identified and selected as talented football players. It is thus interesting to investigate how the players themselves understand both their own talent and talent in general. Do they perceive talent in terms of being static, meaning hereditary, or in terms of being dynamic, meaning the result of targeted training over time? When asked directly about the importance of heritage, two of the girls gave the following answers:

“I do not think there is much (heredity)” (G2).

“One is not born with a talent… One gets better; we are after all not born with skills” (G4).

In other words, there was consensus among the girls in the two focus groups, indicating a weak link between talent and heritage. Instead they perceive talent as something they must train for, and thus have a dynamic perception of skill:

“(…) We have worked for it, many believe that, or that, many may say that a talent, you’ve got it, you’ve inherited things and stuff, but I think that we have also trained for it, we want to accomplish something, it does not come by itself” (G3).

Among males, however, there is a more widespread belief that talent is something innate:

“There are many that are good from the start somehow. That just has it in him” (B5).

“One cannot start without having good genes. You cannot start as completely miserable” (B3).

The boys in the study connect their talent to genes and something they just “have in them”, and thus seem to have a more static perception of the skill concept. It may therefore seem that boys partially agree with the basic assumptions that are often associated with talent, with talent being understood as hereditary [2]. This does not necessarily imply that one is born to be a footballer, but talent can just as easily be related to inherited ability to handle high amounts of training or mental strength:

“It is possible to have a talent for training too. They just train themselves good” (B2).

“Either you have the will and the mental strength or you do not” (B5).

Results show a clear gender dimension in how players perceive their talent. Girls have a more dynamic perception of talent, which basically means that talent is a result of training [5]. Boys, on the other hand, have a more static perception of talent, understanding talent to be largely inherited. The static understanding of talent holds true for both football talent and training talent, leading to an understanding of targeted training having a limited effect. However, this does not imply that talent is taken for granted for the foreseeable future. As some of the players stated:

“The talent can stop anytime. Perhaps at this time, now (15–16 years)” (B2).

“Many people say that we float on a talent, but that it’s maybe at a young age” (G1).

Regardless of the importance of the genetic component of talent, several players highlight the importance of developing skills, and not only relying on their current skills, especially since they consider their current age as a turning point in their potential professional career. A potential explanation for the players’ difficulties in defining which skills they should develop could be seen in line with the difficulties even top-level coaches have when asked to define which criteria they use to identify the most talented players [7].

A talent is something you can “lose”, according to several of the players. One example mentioned by one of the players was the importance of training, and the fact that players not defined as talented could have a higher training load and thus achieve a higher skill level based on training. According to Ericsson and his colleagues [18], players’ skills come as a direct result of their training load, and could thus explain why some players “catch up” in terms of skill level. Even so, training load has not been found to be a significant indicator to predict future skills [19]. Overall, several players describe their talent as something short-term, confined to a young age, that can be lost if you take a breather in your development as a player. On one hand, this is in agreement with the dynamic perception of talent [5]. On the other hand, it does not seem like the players think they can retrieve a talent if first lost, which fits better into a static perception of talent.

Compared with the girls in this study and earlier studies on elite coaches [7], the boys in this study appear to embrace a more static perception of talent. One explanation could be that boys are part of a tougher competition for resources. Being among the selected and getting access to these resources has been found to be essential for young players’ development [26], and provides obvious advantages for future development [3]. Coaches that focus on results and achievement among young players rely on a static perception of talent and utilize present performance levels and physical properties as the basis for identifying and selecting the best talent [24]. This could obviously affect the player’s perception of his or her talent as a static condition. Coaches who are concerned about short-term results, in terms of winning football matches and giving playing time to the best players, will favor the best players at any given time.

### 4.2. Highly Specified or General Skills

In accordance with the literature of talent development [22], most talented players would agree to highlight training load as an essential factor in talent development. Even so, the importance of training quality and which skills one should develop is a more complicated question. One of the basic assumptions regarding talent focuses on talent as being domain-specific [2], indicating the importance of developing highly specified skills. When the players were asked whether they should develop highly specified skills or more general skills, there was dissensus. Similar to how players perceive talent and how to develop skills, the gender dimension was present with boys clearly prioritizing highly specified skills:

“I have always learned that if there is one thing I am good at I should focus on training more on that. To get even better at it” (B2).

“It is also important to have one very good skill” (B3).

“You can always get better at what you are good at. So it is important to work on it too” (B1).

A few of the boys were, however, somewhat more diffuse about whether they should pursue more specified skills or not.

“Mainly focus on what I do best and take what I’m worst at eventually” (B4).

“Both really. Develop what you are good at and what you are bad at actually” (B5).

As the quotes above show, all of the boys did not prioritize highly specified skills; rather, it seems like some of them prioritized both types of skills. The girls were even more concerned about finding the balance between general and specified skills, and some were quite clear that they prioritize general skills:

“I would have developed what I’m bad at. And continue to maintain what I am good at” (G5).

“I would have done both. But mostly what I’m bad at” (G4).

“It’s important to practice what you might not be so good at. So you get good at it. But do not stop practicing what you are good at either. It’s good that you have the advantage in something. And continue to work at it” (G2).

Overall, results show that boys prioritize highly specified skills rather than general skills. Girls in this sample seem to distribute equal value to general and highly specific skills. Asked to prioritize, several of the girls would give priority to developing general skills. Taken together, results in this study show a relationship between gender, perception of talent, and prioritization of highly specified or general skills. The girls have a more dynamic understanding of talent and prioritize general skills to a greater extent, confirming the perception of compensating for deficiencies in one area by strength in others [20]. One way of explaining these results is through combining what we know about coaches often utilizing present performance levels and physical properties as the basis for identifying and selecting the best talent [24], together with the tendency of selecting talented players by an early birth month, the relative age effect [15,16,17]. Since the relative age effect appears less pronounced for female players [14], we suggest that girls are part of a selection system that allows them to develop a more dynamic perception of talent compared with the boys.

Another potential explanation to why the boys focus on specific skills relates to Ericsson and his colleagues [18], understanding deliberate practice as a success factor in talent development. Highlighting the importance of deliberate practice could indicate specialization according to both playing position and developing specified skills, which girls in this study seem to deem less important compared to the boys. Even though the coaches’ role in talent identification and development is obvious [23,24,25], the players’ perception of their own talent and their tactics to overcome obstacles [5] is also important for their development. Earlier studies show that coaches have problems defining the criteria by which they identify talented players [7]. This is an obvious obstacle for the talented players if they are uncertain about which skills they should develop to heighten the possibility of becoming an elite-level player.

Accepting that boys have a more static perception of talent and are part of a selection system that relies more heavily on basic assumptions regarding talent development [8,11] has some interesting implications. First of all, coaches should be aware of the possible vulnerability following players’ static perception of talent. Such players would typically explain a lack of success through lacking abilities and might not see the point of making an effort to improve their skills through training. Second, locking onto highly specified skills, instead of finding a balance between specified and general skills, might make for a less optimal preparation for the changing demands young players meet when moving through the different levels of play on their way to high-level football.

### 4.3. Limitations of the Study and Future Research

The relatively small number of participants is an obvious limitation of this study. Even if the recruited players were included based on the criteria of selection to a district, regional or age-specific national team during the latest 12 months, these players would mostly be representative of the regional football district. The limited number of topics in this study could also be seen as a limitation, since a broader span of topics could have given a better understanding of the players’ perceived talent and abilities. The limitations of focus group interviews regarding the depth of information should also be mentioned [29]. It is, for instance, not exactly clear what the participants think are highly specified and general skills. It is reasonable to assume that the distinction would differ from player to player, and that semi-structured interviews would be beneficial in future research. Future research should also investigate how players from other districts perceive their own talent compared to the current study. Furthermore, it would also be of interest to investigate players with a lower skill level, not yet regarded as having talent, and how they perceive their talent and skill development. One could hypothesize a relationship between the players’ perceived talent and selection, indicating that a selection could be seen as a confirmation of ability, thereby showing that these players perceive their abilities to be higher than those of non-selected talented players.

## 5. Conclusions

The objective of the article is to investigate how boys and girls perceive their talent and to discuss how various perceptions influence coaching practice in talent development. The first objective of this study was to investigate if players have a static or dynamic perception of their own talent. Results show that talented girl footballers experience a weak link between talent and heritage, and have a dynamic perception of the skill concept. Among talented boy footballers there exists a widespread perception of talent to be something innate. The boys in the sample connect talent to genes and something that players just “*have in them”*, and seem to have a more static perception of the skill concept, agreeing to many of the basic assumptions often associated with talent [2]. Even so, hard work is described as important. Players understand their own talent as having a kind of breaking point, being able to “surf” on their talent in younger years, but that their peers will catch up if they take a breather in developing as a player. This perception may be described as a dynamic perception of talent in accordance with Abbott and Collins [5]. The second objective was to investigate if the players consider specific or general skills most important in their skill development. Results show that the girls were more concerned about finding the balance between highly specified and general skills, and clearly give priority to general skills. The boys, however, were keen to develop highly specified skills, although some of the boys point out the importance of having both specified and general skills.

## References

[1] Simonton J.L. (1999). Talent and its development: An emergenic and epigenetic model. Psychol. Rev..

[2] Durand-Bush N., Salmela J.H., Singer R.N., Hausenblas H.A., Janelle C.M. (2001). The development of talent in sport. Handbook of Sport Psychology.

[3] Ashworth J., Heyndels B. (2007). Selection bias and peer effects in team sports: The effect of age grouping on earnings of German soccer players. J. Sports Econ..

[4] Larsen C.H., Alfermann D., Henriksen K., Christensen M.K. (2013). Successful talent development in soccer: The characteristics of the environment. Sport Exerc. Perform. Psychol..

[5] Abbott A., Collins D. (2002). A theoretical and empirical analysis of a “state of the art”talent identification model. High Abil. Stud..

[6] Miller P.K., Cronin C., Baker G. (2015). Nurture, nature and some very dubious social skills: An interpretative phenomenological analysis of talent identification practices in elite English youth soccer. Qual. Res. Sport Exerc. Health..

[7] Sæther S.A. Talent Identification in Soccer. What do Coaches Look for?. http://idrottsforum.org/wp-content/uploads/2014/03/saether140319.pdf.

[8] Helsen W.F., Hodges N.J., Winckel J.V., Starkes J.L. (2000). The roles of talent, physical precocity and practice in the development of soccer expertise. J. Sports Sci..

[9] Reilly T., Williams A.M., Nevill A., Franks A. (2000). A multidisciplinary approach to talent identification in soccer. J. Sports Sci..

[10] Williams A.M., Reilly T. (2000). Talent identification and development in soccer. J. Sports Sci..

[11] Abbott A., Collins D. (2004). Eliminating the dichotomy between theory and practice in talent identification and development: Considering the role of psychology. J. Sport Sci..

[12] Gledhill A., Harwood C. A Systematic Review of Psychological Factors Associated with Talent Development in Soccer. Proceedings of the 13th FEPSAC European Congress of Sport Psychology.

[13] Van Yperen N.W. (2009). Why some make it and others do not: Identifying psychological factors that predict career success in professional adult soccer. Sport Psychol..

[14] Schorer J., Cobley S., Bilsch D., Brautigam H., Baker J. (2009). Influences of competition level, gender, player nationality, career stage and playing position on relative age effects. Scand. J. Med. Sci. Sports.

[15] Brewer J., Balsom P., Davis J. (1995). Seasonal birth distribution amongst European soccer players. Sports Exerc. Inj..

[16] Helsen W.F., Starkes J.L., Hodges N.J. (1998). Team sports and the theory of deliberate practice. J. Sports Exerc. Psychol..

[17] Simmons C., Paull G.C. (2001). Season-of-birth in association football. J. Sports Sci..

[18] Ericsson K.A., Krampe R., Tesch-Römer C. (1993). The role of deliberate practice in the acquisition of expert performance. Psychol. Rev..

[19] Cote J., Fraser-Thomas J., Farrow D., Baker J., MacMahon C. (2008). Play, practice and athlete development. Developing Sport Expertise.

[20] Meylan C., Cronin J., Oliver J., Hughes M. (2010). Reviews: Talent identification in soccer: The role of maturity status on physical, physiological and technical characteristics. Int. J. Sports Sci. Coach..

[21] Stølen T., Chamari K., Castagna C., Wisløff U. (2005). Physiology of soccer: An update. Sports Med..

[22] Haugaasen M., Jordet G. (2012). Developing football expertise: A football-specific research review. Int. Rev. Sport Exerc. Psychol..

[23] Carlson R. (1991). Vägen til landslaget. En retrospektiv Studie av Framgångsrika Ungdomar i sju Idrottar.

[24] Gagné F., Heller K.A., Mönks F.J., Sternberg R.J., Subotnik R. (2000). Understanding the complex choreography of talent development through DMGT-based analysis. International Handbook for Research on Giftedness and Talent.

[25] Martindale R.J.J., Collins D., Abraham A. (2007). Effective talent development: The elite coach perspective in UK sport. J. Appl. Sport Psychol..

[26] Christensen M.K., Sørensen J.K. (2009). Sport or school? Dreams and dilemmas for talented young Danish football players. Eur. Phys. Educ. Rev..

[27] Dowling F.N. (2001). Naratives about Young Men and Masculinities in Organised Sport in Norway. Sport Educ. Soc..

[28] Berg B.L., Lune H. (2012). Qualitative Research Methods for the Social Sciences.

[29] Lune H., Enrique P., Koppel R. (2009). Perspectives in Social Research Methods and Analysis. A Reader for Sociology.

